# Third molar position after completion of orthodontic treatment: a prospective follow-up

**DOI:** 10.1259/dmfr.20220432

**Published:** 2023-05-02

**Authors:** Charlotte Butaye, Annelie Miclotte, Giacomo Begnoni, Zuodong Zhao, Chen Zong, Guy Willems, Anna Verdonck, Reinhilde Jacobs, Maria Cadenas de Llano-Pérula

**Affiliations:** 1 Department of Oral Health Sciences-Orthodontics, KU Leuven and Dentistry, University Hospitals Leuven, Kapucijnenvoer, Leuven, Belgium; 2 OMFS IMPATH, Department of Imaging & Pathology, Faculty of Medicine, University Leuven & Maxillofacial Surgery, University Hospitals Leuven, Kapucijnenvoer, Leuven, Belgium; 3 Department of Dental Medicine, Karolinska Institutet, Stockholm, Sweden

**Keywords:** Orthodontics, Third molars, Tooth Impaction, Cervical Headgear, Premolar extraction

## Abstract

**Objectives:**

To prospectively follow up a previously reported sample, analyzing (1) changes in third molar (M3) position after completion of 2 different types of orthodontic treatment: (2) non-extraction treatment with (HG) *vs* without cervical headgear (non-HG) and (3) first or second premolar extractions (PM1-2) compared to a non-extraction group (NE).

**Methods:**

A total of 474 patients were prospectively followed up. Panoramic radiographs were taken pre- (T1), post-treatment (T2) and at follow-up (T3). T3 records (a mean of three years after treatment) were available for 135 (HG *vs* non-HG) and 134 patients (PM1-2 *vs* NE), respectively. Angulation, vertical position, relation with the mandibular canal and mineralization status of M3 at T2 and T3 were statistically compared.

**Results:**

The HG group presented more M3 with ideal vertical orientation at T3. In NE-cases, further improvement in angulation and orientation can be expected after debonding, as well as a deterioration in the relationship with the mandibular canal. Extractions accelerated upper M3 vertical eruption and PM2 extractions led to long-term larger lower retromolar spaces.

**Conclusions:**

The use of cervical headgear increased upper M3 uprighting three years after debonding, while little changes in M3 position were found after orthodontic treatment with extractions. However, PM2 extractions led to larger retromolar spaces and better M3 angulation in the long term.

## Introduction

Third molar (M3) impaction is an important clinical problem in dentistry and maxillofacial surgery. According to a recent meta-analysis,^1^ the average worldwide rate of M3 impaction is 24,40%. Impacted M3 can cause pericoronitis, decay or root damage to the second molar and development of cysts and tumors. Unsurprisingly, the removal of impacted M3 is one of the most common surgical procedures performed by oromaxillofacial surgeons.^2^ Nevertheless, preventive removal of M3, often at the germination state, remains highly debated, mostly due to the risk of complications and the high costs for social health insurances.

The effect of orthodontic treatment on M3 position has been extensively studied in related literature, since insufficient retromolar space has been suggested to be a key factor in M3 impaction.^3^ Orthopedic forces applied to the maxilla in combination with orthodontic distalization of the upper molars result in a reduction of the retromolar space.^4^ Orthodontic extractions on the other hand have been shown to have a positive effect on the retromolar space.^5–7^ However, most available studies only include short follow-up periods, while long-term longitudinal study designs are scarce.^4,6,8–11^ Nevertheless, most orthodontic treatments are performed in patients whose M3 are not yet fully developed and can therefore influence their further eruption pattern. Increasing the knowledge regarding the expected changes of M3 in the years after completion of orthodontic treatment could offer the clinician valuable guidelines regarding treatment planning and possibly early referral to the oromaxillofacial surgeon.

The aims of the present study were to analyze the further change in position of M3 of a previously reported sample (1) on average three years after completion of orthodontic treatment, and (2) to compare M3 position among patients treated with or without cervical headgear and (3) with and without premolar extractions.

## Methods and materials

The protocol of this study was approved by the medical ethical committee of University Hospitals Leuven and KU Leuven University and the study was conducted in accordance with the Helsinki Declaration.

### Subject recruitment

A total of 474 growing patients receiving treatment at the Department of Orthodontics of University Hospitals Leuven and with radiographic evidence of at least one M3 were prospectively followed up for an average of 6 years. Patients with craniofacial disorders or missing teeth, due to agenesis or extraction, were excluded.

Patients were screened before treatment, when a first panoramic radiograph was taken (T1) and received the following treatment types, which were used to further subdivided them into: cervical headgear (HG) *vs* noheadgear (non-HG), and (upper and/or lower) first (PM1) or second premolar (PM2) extractions *vs* non-extractions (NE). Afterwards, fixed modified edgewise appliances were used in all patients. All participants finished their treatment between January 2008 and December 2014. After comprehensive orthodontic treatment a second panoramic radiograph was taken (T2). The details of the sample at T1, the performed treatment and its effect on M3 (T1-T2) have been previously reported, and the mean M1 distalization in HG group was 0.9 ± 2.3 mm, measured by the distance from pterygoid vertical (PTV) to the distal surface of the upper M1 crown (PTV-M1).^4,6^

### Methods

Patients were prospectively followed up by recall visits at 1, 3, and 6 months, and 1, 2, 5, and 10 years after debonding of orthodontic fixed appliances. At these consultations, the stability of the achieved occlusion and the state of the retainers were clinically evaluated. An average of three years after T2, a third panoramic radiograph was taken (T3).

The following parameters were evaluated on the panoramic images taken at T2 and T3: First, the angle between the long axis of the second and third molar (M3^M2) was measured. Two reference points defined this axis: the center of the mesio-distal crown-width and the molar furcation. In case the roots were not formed yet, the most apical point of the pulp cavity of the M3 was taken, and the axis was drawn perpendicular to the occlusal surface of the crown. Mesio-angular angulation of the M3 in the lower jaw results in a positive angle measurement and disto-angular angulation results in a negative value. In the upper jaw it is just the opposite.^4,6^ Secondly, the orientation of the third to the second molar was scored by the classifications suggested by Archer (upper molars)^12^ and Winter (lower molars).^13^ Only fully erupted second molars were considered. Moreover, the vertical eruption level of M3 was scored using the Archer classification for upper molars and Pell and Gregory’s vertical classification for lower molars. The Pell and Gregory horizontal classification was used to determine the eruption space for lower M3.^14^ Additionally, The Demirjian classification was applied to assess the mineralization status.^15^ Finally, a shortened version of the Whaites classification was developed to estimate the risk of a close relationship between the roots of M3 and the mandibular canal. The following radiographic landmarks were indicative for a close relationship between the mandibular canal and the roots of the wisdom teeth: loss or narrowing of tramlines, alteration of direction of the inferior canal at the root apex and a radiolucent band across the roots.^16^

All measurements were performed on panoramic radiographs generated by a Veraview device, Morita (Kyoto, Japan) or a Cranex Tome, Soredex (Tuusula, Finland) by two trained and calibrated observers (M.A. and B.C.) under standard viewing conditions. Measurements were performed on IMPAX software (Agfa healthcare, Mortsel, Belgium).

### Statistical analysis

Intra- and interobserver reliability was analyzed using SPSS software (version 24, IBM, Chicago, USA). For the comparison between patients treated with and without cervical headgear or premolar extractions, Fisher’s exact tests and Mann-Whitney U tests were used for nominal and continuous variables, respectively. To evaluate group differences or changes over time, a linear model for longitudinal measurements was used, modelling an unstructured covariance matrix to account for repeated measurements in time, and a random intercept to account for measuring different teeth within subjects. A similar approach was followed for the ordinal data and the nominal scores, but the linear model was replaced with a logistic regression model using generalized estimating equations (GEE) to handle the correlation between both time points and teeth. All tests were performed on a two-sided 5% significance level. Analyses were performed using SAS software (version 9.4 of the SAS System for Windows).

## Results

The descriptive data are summarized in Table 1. A total of 474 orthodontically treated patients were prospectively followed. From this, 116 patients were included both in the non-HG and NE group. Follow-up (T3) records were available for 224 patients. Most dropouts are related to patient’s absence on follow-up appointments. In the minority of cases, there was no need for wisdom tooth removal, or the patient was referred for removal immediately after debonding. Of the final sample, 81 patients were treated with cervical headgear (HG) and 54 patients without cervical headgear (non-HG). Their follow-up period after the end of orthodontic treatment was on average 3.1 years. Further, a follow-up panoramic radiograph was available for 36 patients treated with extractions (PM1-2) and 98 patients treated without extractions (NE) with a mean follow-up period after debonding of 3 years. Within the PM1-2 group, 10 patients had extractions only in the upper jaw, three only in the lower jaw and 23 patients in both jaws. Intra- and interobserver reliability were excellent: interclass correlation coefficients were 0.99 and 0.97, respectively.

**Table 1.** Sample distribution by gender, age and treatment

		All patients	Non-Headgear	Headgear	*p*-value	Non-Extraction	Extraction (*N* = 78) (PM1 = 56, PM2 = 35)	*p*-value
Gender (n/N (%))	Male	227/474 (47.9)	61/134 (45.5)	79/160 (49.4)	0.558	103/218 (47.3)	37/78 (47.4)	1.000
	Female	247/474 (52.1)	73/134 (54.5)	81/160 (50.6)		115/218 (52.8)	41/78 (52.6)	
								
								
Age post-treatment (T2) (years)	N	474	134	160		218	78	
	Mean	15.6	15.2	15.5	0.059	15.5	15.9	0.143
	Range	11.8–21.3	11.8–17.8	12–19.4		12.9–20.3	12.5–21.3	
								
								
Age follow-up (T3) (years)	N	224	54	81		98	36	
	Mean	18.5	18.4	18.2	0.829	18.3	19.3	0.032
	Range	5–25.4	15.1–23.6	15.2–25.4		15–23.3	15.5–24	
								
								
Follow-up period (years)	Mean	3.1	3.5	2.9	0.062	3	3.3	0.416
	Range	0.5–10.4	0.7–10.5	0.5–8.4		0.7–8.3	0.9–5.9	
								
(n/N (%))	<3y	135/224 (60.3)	28/54 (51.9%)	50/81 (61.7%)	0.462	61/98 (55.6%)	81/134 (60.5%)	0.677
	3-5y	53/224 (23.7)	15/54 (27.8%)	20/81 (24.7%)		10/98 (27.8%)	31/134 (23.1%)	
	>5y	36/224 (16.1)	11/54 (20.4%)	11/81 (13.6%)		6/98 (16.7%)	22/134 (16.4%)	

PM1: first premolar extraction, PM2: second premolar extraction. A total of 116 patients were included both in the Non-headgear and Non-extraction group.

### Effect of orthodontic treatment (all groups) on M3 three years after completion of orthodontic treatment Tables 2 and 3

**Table 2.** General outcome information for Upper third molar position 3 years after the end of orthodontic treatment

Measurement	M3^M2 (upper jaw)	Measurement	Archer (orientation)	Measurement	Archer (vertical)	Measurement	Demirijan
End of treatment							
Mean (°)	14.22	Stage 1 (%)	11.6	Stage 1, 2 and 3 (%)	14.6	Stage 1, 2, 3 and 4 (%)	30.4
Median (°)	13.45	Stage 2 (%)	21.3	Stage 4 and 5 (%)	85.4	Stage 5, 6, 7 and 8 (%)	69.6
IQR (°)	(3.22; 23.44)	Stage 3 (%)	66.1				
		Stages 4 to 6 (%)	1				
Follow-up period							
Mean (°)	7.84	Stage 1 (%)	10	Stage 1, 2 and 3 (%)	54.2	Stage 1, 2, 3 and 4 (%)	5.8
Median (°)	9.30	Stage 2 (%)	6	Stage 4 and 5 (%)	45.8	Stage 5, 6, 7 and 8 (%)	94.2
IQR (°)	(0.30; 16.80)	Stage 3 (%)	83.1				
		Stages 4 to 6 (%)	1				
Change T2 *vs* T3 (p- value)							
^a^<.0001^*^		Stage 1	^b^0.390	^b^<.0001^*^		^b^<.0001^*^	
		Stage 2	^b^<.0001^*^				
		Stage 3	^b^<.0001^*^				

M2^M3: angle between the long axis of second and third molar. ^a^ *p*-values based on linear model without correction of confounders.^b^logistic regression model without correction of confounders. ^*^ *p*-values smaller than 0.05 are considered significant.

**Table 3.** General outcome information for Lower third molar position 3 years after the end of orthodontic treatment

Measurement	M3^M2 (lower jaw)	Measurement	Winter (orientation)	Measurement	PGV	Measurement	PGH	Measurement	Whaites
End of treatment									
Mean (°)	26.03	Stage 1 (%)	19.1	PGV-1 (%)	3.1	PGH-1 (%)	31.1	positive relationship (%)	39.2
Median (°)	26.16	Stage 2 (%)	1.6	PGV-2 (%)	11.8	PGH-2 (%)	59.7		
IQR (°)	(18.27; 33.31)	Stage 4 (%)	79	PGV-3 (%)	85.1	PGH-3 (%)	9.2		
		Stages 3, 5, 6 (%)	0.2						
Follow-up period									
Mean (°)	26.26	Stage 1 (%)	44.7	PGV-1 (%)	16.5	PGH-1 (%)	43	Positive relationship (%)	52.3
Median (°)	26.70	Stage 2 (%)	1.7	PGV-2 (%)	25.7	PGH-2 (%)	44.7		
IQR (°)	(13.40; 36.00)	Stage 4 (%)	51.1	PGV-3 (%)	57.8	PGH-3 (%)	12.3		
		Stages 3, 5, 6 (%)	2.5						
Change T2 *vs* T3 (p- value)									
^a^0.656		Stage 1	^b^<.0001^*^	^b^<.0001^*^		PGH-1	0.004^*^	^b^0.001^*^	
		Stage 4	^b^<.0001^*^			PGH-2	0.001^*^		
						PGH-3	0.180		

M2^M3: angle between the long axis of second and third molar; PGV: Pell & Gregory vertical classification; PGH: Pell & Gregory horizontal classification. ^a^p-values based on linear model without correction of confounders.^b^logistic regression model without correction of confounders. ^*^ *p*-values smaller than 0.05 are considered significant.

Significant further uprighting (M3^two and Archer) of upper M3 can be expected after orthodontic treatment. Although the change in angulation (M3^2) of lower M3 stagnated after debonding, the orientation (Winter) significantly improved. Finally, a significant enlargement of the lower retromolar space was observed three years after completion of orthodontic treatment.

### Effect of cervical headgear on upper M3 three years after completion of orthodontic treatment (HG vs. non-HG, Table 4)

**Table 4.** General outcome information and comparison between Upper third molar position 3 years after the end of orthodontic treatment with Headgear *vs* Non-headgear

Measurement	M3^M2 (upper jaw)		Measurement	Archer (orientation)		Measurement	Archer (vertical)		Measurement	Demirjian		
	Non-HG	HG		Non-HG	HG		Non-HG	HG		Non-HG		HG
End of treatment												
Mean (°)	14.87	13.85	Stage 1 (%)	13.8	13.5	Stages 1 to 3 (%)	8.6	12.3	Stages 1 to 4 (%)	53.6		45.2
Median (°)	13.06	12.17	Stage 2 (%)	20.5	23.9	Stages 4 and 5 (%)	91.4	87.7	Stages 5 to 8 (%)	46.4		54.8
IQR (°)	(2.91; 13.84)	(3.76; 22.09)	Stage 3 (%)	63.8	61.6							
			Stages 4 to 6 (%)	1.9	0.9							
Follow up period												
Mean (°)	5.05	7.90	Stage 1 (%)	18.5	6.3	Stages 1 to 3 (%)	50.9	49.1	Stages 1 to 4 (%)	4.4	10.7	
Median (°)	6.95	10.10	Stage 2 (%)	3.7	4.4	Stages 4 and 5 (%)	49.1	50.9	Stages 5 to 8 (%)	95.6	89.3	
IQR (°)	(−3.10; 15.50)	(0.90; 16.40)	Stage 3 (%)	75.9	89.3							
			Stages 4 to 6 (%)	1.9	0							
Change (*p* value HG *vs* non-HG)												
	^a^0.020^*^		Stage 1	^b^0.017^*^			^b^0.507			^b^0.416		
			Stage 2	^b^0.984								
			Stage 3	^b^0.010^*^								

a M2^M3: angle between the long axis of second and third molar.^1^ *p*-values based on linear model without correction of confounders.^2^ logistic regression model without correction of confounders. ^*^ *p*-values smaller than 0.05 are considered significant.

M3 angulation (M3^M2) improved during follow-up in both groups (*p* < .0001), and significantly more the non-HG group. Between T2 and T3, M3 with a mesio-angular and disto-angular orientation (Archer) uprighted in the HG group (*p* = 0.010; <.0001), leading to an increase in M3 with a vertical orientation (*p* < .0001). In the non-HG group contrarily, only a decrease for Stage two was noticed (*p* = 0.003). As a result, significantly more patients in the HG group had an ideal vertical orientation (*p* = 0.011) and significantly less patients had an unfavorable mesio-angular orientation (*p* = 0.008) at T3. Further vertical intraosseous eruption of M3 (Archer) was found in both groups (*p* < .0001) between T2 and T3, but it did not differ between groups. Finally, the Demirjian analysis revealed a significant but even further development of M3 in both groups (*p* < .0001 and 0.002).

### Effect of premolar extractions on M3 3 years after completion of orthodontic treatment (NE vs. PM1 vs. PM2, Tables 5 and 6)

**Table 5.** General outcome information and comparison between Upper third molar position 3 years after the end of orthodontic treatment with Premolar extraction *vs* Non-extraction

Measurement	M3^M2 (upper jaw)			Measurement	Archer (orientation)			Measurement	Archer (vertical)			Measurement	Demirijan				
	NE	PM1	PM2		NE	PM1	PM2		NE	PM1	PM2		NE		PM1		PM2
End of treatment																	
Mean (°)	14.36	12.98	11.32	Stage 1 (%)	12.5	5	6.3	Stage 1, 2 and 3 (%)	10.4	31.7	34.4	Stage 1, 2, 3 and 4 (%)	30.6	24		22.8	
Median (°)	12.92	16.61	13.53	Stage 2 (%)	20.1	12.9	12.5	Stage 4 and 5 (%)	89.6	68.3	65.6	Stage 5, 6, 7 and 8 (%)	69.4	76		77.2	
IQR (°)	(2.54; 23.53)	(5.60; 25.17)	(2.39; 19.10)	Stage 3 (%)	66	82.2	81.3										
				Stages 4 to 6 (%)	1.4	0	0										
Follow up period																	
Mean (°)	7.74	7,91	10.41	Stage 1 (%)	12	17.3	0	Stage 1, 2 and 3 (%)	51.1	71.2	90.9	Stage 1, 2, 3 and 4 (%)	5.7	2.5		5.7	
Median (°)	8.9	9.8	9.6	Stage 2 (%)	8.2	5.8	0	Stage 4 and 5 (%)	48.9	28.9	9.1	Stage 5, 6, 7 and 8 (%)	94.3	97.5		94.3	
IQR (°)	(0.13; 15.95)	(−0.15; 18.30)	(4.10; 22.30)	Stage 3 (%)	77.7	76.9	100										
				Stages 4 to 6 (%)	2.2	0	0										
Change (*p* value)					Stage 1	Stage 2	Stage 3										
NE *vs* PM1	^a^0.537			NE *vs* PM1	^b^0.042^*^	^b^0.829	^b^0.065	NE *vs* PM1	^b^0.224			NE *vs* PM1	^b^0.556				
NE *vs* PM2	^a^0.402			NE *vs* PM2				NE *vs* PM2	^b^0.378			NE *vs* PM2	^b^0.346				
PM1 *vs* PM2	^a^0.785			PM1 *vs* PM2				PM1 *vs* PM2	^b^0.152			PM1 *vs* PM2	^b^0.194				
																	

NE: non-extraction; PM1: first premolar extraction; PM2: second premolar extraction; M2^M3: angle between the long axis of second and third molar. ^a^ *p*-values based on linear model without correction of confounders. ^b^logistic regression model without correction of confounders. ^*^ *p*-values smaller than 0.05 are considered significant.

**Table 6.** General outcome information and comparison between Lower third molar position 3 years after the end of orthodontic treatment with Premolar extraction *vs* Non- extraction

Measurement	M3^M2 (lower jaw)			Measurement	Winter (orientation)				Measurement	PGV			Measurement	PGH			Measurement	Whaites		
	NE	PM1	PM2		NE	PM1		PM2		NE	PM1	PM2		NE	PM1	PM2		NE	PM1	PM2
End of treatment																				
Mean (°)	26.35	26.64	23.19	Stage 1 (%)	16.6	28.6		30	PGV-1 (%)	1.4	16.3	5	PGH-1 (%)	23	46.9	76.7	positive relationship (%)	40.2	36.7	33.3
Median (°)	26.45	27.16	25.07	Stage 2 (%)	1.4	4.1		1.7	PGV-2 (%)	9	10.2	33.3	PGH-2 (%)	66	49	23.3				
IQR (°)	(18.97; 33.15)	(16.56; 35.61)	13.59; 31.24)	Stage 4 (%)	81.8	67.4		68.3	PGV-3 (%)	89.7	73.5	61.7	PGH-3 (%)	11	4.1	0				
				Stages 3, 5, 6 (%)	0.2	0		0												
Follow up period																				
Mean (°)	26.57	30.1	19.53	Stage 1 (%)	45.2	33.3		54.2	PGV-1 (%)	14.5	29.6	16.7	PGH-1 (%)	37.6	48	79.2	Positive relationship (%)	51.1	59.3	54.2
Median (°)	26.85	32	21	Stage 2 (%)	2.2	0		0	PGV-2 (%)	28	11.1	25	PGH-2 (%)	48.9	36	20.8				
IQR (°)	(13.90; 37.30)	(12.00; 45.50)	(10.75; 30.65)	Stage 4 (%)	50	63		45.8	PGV-3 (%)	57.5	59.3	58.3	PGH-3 (%)	13.4	16	0				
				Stages 3, 5, 6 (%)	2.7	3.7		0												
Change (*p* value)																				
					Stage 1		Stage 4													
NE *vs* PM1	^a^0.966			NE *vs* PM1	^b^0.037^*^		^b^0.028^*^		NE *vs* PM1	^b^0.133			NE *vs* PM1	0.195			NE *vs* PM1	^b^0.333		
NE *vs* PM2	^a^0.168			NE *vs* PM2	^b^0.488		^b^0.311		NE *vs* PM2	^b^0.006^*^			NE *vs* PM2	0.693			NE *vs* PM2	^b^0.447		
PM1 *vs* PM2	^a^0.350			PM1 *vs* PM2	^b^0.303		^b^0.331		PM1 *vs* PM2	^b^0.542			PM1 *vs* PM2	0.689			PM1 *vs* PM2	^b^0.934		
																				

NE: non-extraction; PM1: first premolar extraction; PM2: second premolar extraction; M2^M3: angle between the long axis of second and third molar; PGV: Pell & Gregory vertical classification; PGH: Pell & Gregory horizontal classification.^a^ *p*-values based on linear model without correction of confounders. ^b^logistic regression model without correction of confounders. ^*^ *p*-values smaller than 0.05 are considered significant.

In the three-year period after the end of NE treatment, significant further uprighting (M3^M2) of upper M3 was observed (*p* < .0001). No significant differences could be found between groups at T3 in the upper jaw, in contrast to lower, where M3 were significantly more uprighted when PM2 were extracted compared to NE cases (*p* = 0.029). Over time, significantly more upper M3 with mesio-angular orientation (Archer) were seen in the PM1 group (*p* = 0,029), in contrast to the NE group, where a significant further uprighting (decrease Stage 2, increase Stage 3) (*p* = 0.001; *p* = 0.013) was seen. Fisher exact tests performed on the PM2 group, where all M3 had a vertical orientation at T3, showed no significant change over time, nor a group difference at either time point. In the lower jaw, a significant increase for M3 with vertical orientation (*p* < .0001) and a significant decrease for M3 with mesio-angular orientation (*p* < .0001) (Winter) was seen during follow-up in the NE group. For both scores, the difference in change over time between the PM1 and the NE group was significant.

Patients treated without extractions presented upper M3 with significantly higher vertical positions (Archer) compared to patients treated with extractions at both time points (T2: *vs* PM1 and PM2: p= <.0001; *vs* PM2: *p* = 0.001; T3: *vs* PM1 and PM2: *p* = 0.019; *p* = 0.004), while no significant differences were detected between the two extraction groups at either time point. The odds of having a higher score in the Pell and Gregory vertical classification were significantly higher for NE patients at T2 ( *vs* PM1: *p* = 0.009; *vs* PM2: p= <.0001), while at T3, no significant differences were observed between groups. The probability of having a normal lower retromolar space (Pell and Gregory horizontal) was significantly higher in the PM2 group compared to the PM1 group (*p* = 0.009) and in the latter compared to the NE group at T2 (0.010). At T3, this difference is only maintained between PM2 and NE (*p* = 0.004). As for the vertical classification, only in the NE group a significant improvement can be seen between T2 and T3 (p= <.0001; *p* = 0.023). Differences in scores for the Whaites classification between the three groups were non-significant at both time points. Only for the NE group, this (unfavorable) increase over time was found to be significant (*p* = 0.014).

## Discussion

According to a recent systematic review, it is rare for M3 to retain a static angulation over time. However, data to predict further changes in position are not available.^17^ The results of the present study aim to offer the clinician insight into the effect of orthodontic therapy on the position of M3 on average three years after completion of treatment.

### Effect of cervical headgear on upper M3 three years after completion of orthodontic treatment (HG vs. non-HG)

While upper M3 at the end of treatment tend to have a better angulation (M3^M2) in the HG group compared to the non-HG group (although non-significant), this difference seems reversed three years after debonding because of the significantly larger uprighting in the last group. The outcome of the Archer classification is contradictory at first glance because there are significantly more M3 with an ideal vertical orientation in the HG group at T3, while at T2, no difference between both groups was found.^4^ The compromised retromolar space, due to the impeding of the mesial drift of the upper molars and the inhibition of the forward growth of the maxilla by a cervical headgear appliance, forces M3 to take a fully uprighted position more rapidly. The distal tipping of the second molar due to the distalization forces of the cervical headgear may explain the smaller and thus more favorable angular measurements at T2 in this group. The even change over time in vertical position (Archer) of upper M3 in both groups can be explained from a biological point of view. However, it is interesting to note that the use of a cervical headgear appliance does not slow down the descent of upper M3. Abed et al^18^ found a transitory slowing down effect of the use of cervical headgear om the eruption of upper second molar buds, and Nanda and Dandajena^19^ reported delayed eruption of the second molars after prolonged use of cervical headgear.

### Effect of premolar extractions on M3 three years after completion of orthodontic treatment (NE vs. PM1 vs. PM2)

Extractions during treatment did not affect M3 angulation (M3^M2) differently during follow-up. Nevertheless, the greatest improvement can be expected for upper M3 in the NE group and in the lower jaw, PM2 extractions appear to significantly improve the uprighting of M3 in the long term. Only scores 1 (mesio-angular), 2 (disto-angular) and 3 (vertical) of the Archer classification and scores 1 (vertical) and 4 (mesio-angular) of the Winter classification were considered for statistical analysis because of the very small patient numbers in the other groups. All upper M3 had a vertical orientation in PM2 at T3, suggesting that extractions of PM2 lead to a high chance of full uprighting of M3 in the long term. This could not be statistically proven, possibly because of the small sample size of this subgroup (*n* = 11). However, when the orientation of both upper and lower M3 is unfavorable at the end of treatment with PM1-2 extractions, the clinician should not expect significant further improvement and therefore M3 removal should be considered, unlike in NE cases. Further (root) development of M3 and enlargement of the retromolar space during follow-up are possible explanations for the fact that the angulation in the mandible remains unchanged, while the orientation evolves favorably. Much research has already been performed on this topic, with conflicting results. However, most studies focus on the effect during orthodontic treatment itself. A recent systematic review^5^ compares 15 retrospective cohort studies published between 1975 and 2015 investigating this topic, with only six studies having a follow-up after termination of treatment. Artun et al^20^ suggest that premolar extractions favorably affect maxillary M3 angulation. Distal tipping of M3 during active treatment, distal angulation>30°, any mesial angulation relative to the occlusal plane and certainly, mandibular M3 angulated>40° at the end of treatment are risk factors for subsequent impaction. Nance et al.^21^evaluated M3 angulation to the long axis of the second molar on panoramic radiographs in 237 patients aged 14 to 45 years and concluded that only 11% of the M3 with an angulation≥25 degrees and only 3% angled≥35 degrees erupted to the occlusal plane. Lastly, Vranckx et al^22^ stated that mandibular M3 with an initial angulation>27.0° worsen their angulation progressively over time.

PM1-2 extractions accelerate the intraosseous eruption of maxillary M3 in the long term, disregarding which premolar is extracted. While lower M3 in extraction cases present a better vertical position than NE groups at debonding, this difference disappears after three-year follow-up, possibly due to a catch-up movement in the NE group.

Resorption at the anterior border of the ascending ramus during mandibular growth and mesial drift of the posterior teeth during the functional phase of tooth eruption are two important mechanisms in retromolar space development.^3^ PM1-2 extractions initially lead to an enlargement of this space, with this effect being greatest when PM2 are extracted.^6,7^ Mesial displacement of the molars during extraction diastema closure is a logical explanation, since first premolar extractions are often full anchorage cases while second premolar cases are not.^23^ However, after a three-year follow-up period, this difference is only maintained for PM2, consequently leading to an increased chance of supraosseous eruption of M3 in the long term. However, in patients with small retromolar space after extraction treatment, little progression can be expected, in contrast to NE cases. In borderline extraction cases, it is often argued that M3 removal could be avoided by extracting premolars during treatment. Kim et al^24^ investigated the prevalence of M3 impaction in 157 orthodontic cases treated NE or with extraction of 4 premolars with a minimum post-retention period of 10 years. In 80% of the cases, this assumption was true, and for us, 79 and 48% of the patients in the PM2 and PM11 group, respectively, presented sufficient retromolar space at T3 compared to 37,6% in the NE group. Therefore, if preservation of the existing M3 is desired, the clinician may consider extraction of PM2. Prospective cephalometric analysis would have given us the opportunity to objectively analyze the retromolar space and its relation to the patient’s growth pattern, but since no x-rays were taken only for study purposes, this information is not available.

The Whaites classification is suitable for cross-sectional examination of proximity to the alveolar nerve but has its limitations when used in a longitudinal study design: further development of the roots automatically increases the likelihood of a close relationship with the mandibular canal. M3 removal is the most important factor for inferior alveolar nerve (IAN) damage (neurapraxia, axonotmesis, neurotmesis).^25,26^Poort et al found an incidence of 1,2 to 13,4% for temporary IAN damage and 0 to 1,6% for permanent IAN injury.^27^ A close anatomic relationship between the roots of the M3 and the IAN increases the risk of nerve damage. Our results suggest that in NE cases rather than in PM1-2 cases where the M3 is already close to the nerve after the end of treatment, it is advisable to consider M3 removal rather than a wait-and-see attitude to prevent nerve damage. If patients remain in follow-up, surgical M3 removal is highly probable. A recent systematic review showed that retained asymptomatic M3 rarely remain disease-free over time. Caries and periodontal pathology were most frequently observed, especially in partially erupted M3 and mesially inclined mandibular M3.^28^ In order to avoid persistent morbidity and nerve complications, M3 removal before the age of 25 is recommended.^29^ However, a Cochrane review from 2020 concluded that there is insufficient evidence to determine whether asymptomatic, disease-free impacted M3 should be removed or retained. They propose to consider patient values and clinical expertise to guide shared decision-making.^2^

**Figure 1.**
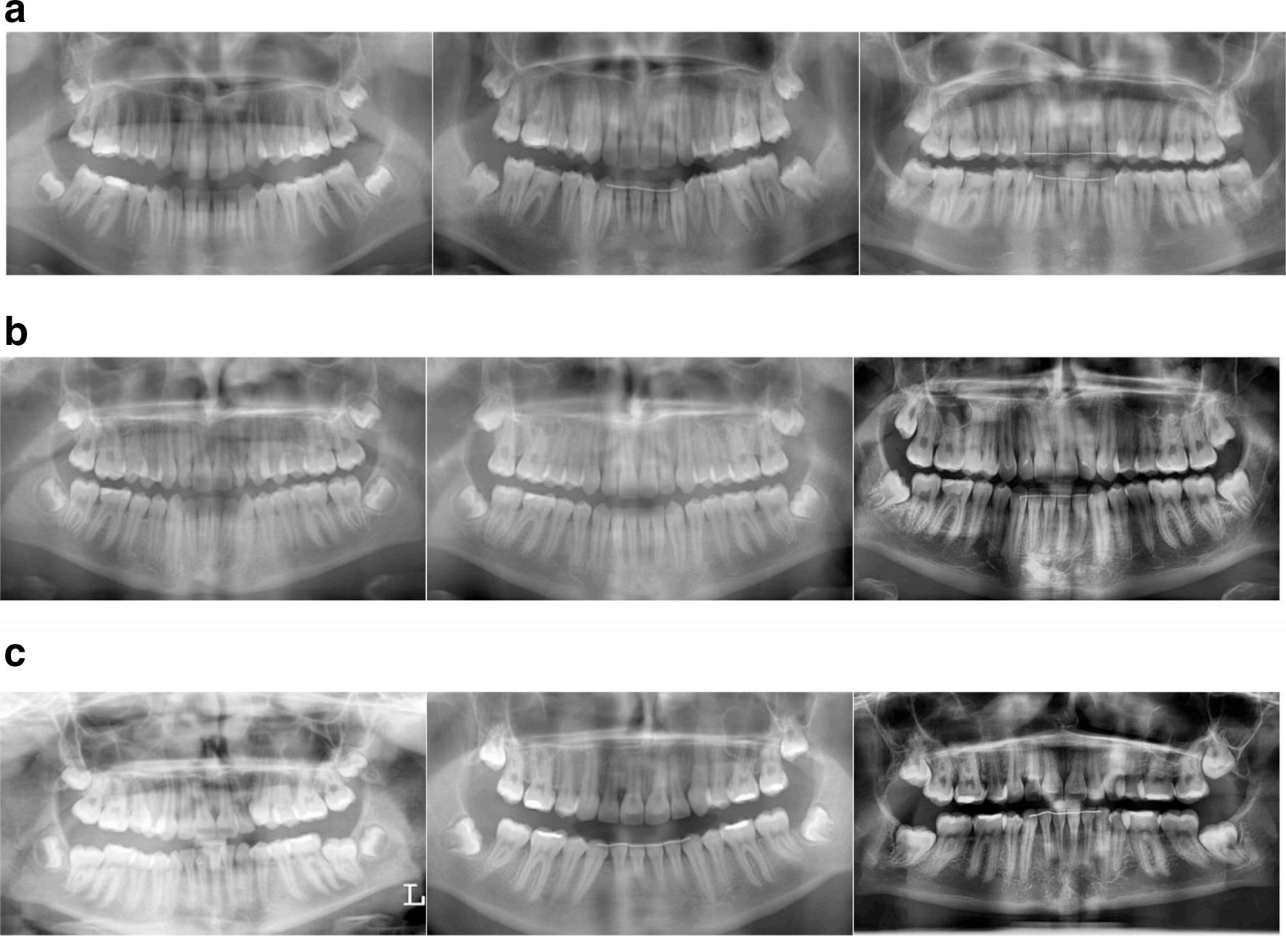
Illustrative cases a. Female patient (24/05/1998) treated with fixed appliances combined with cervical headgear therapy: further uprighting of upper M3 until an ideal vertical orientation at T3 is seen Left T1: 02/07/2010 (M3^M2: 18=22.8°; 28: 16.7°); right T2: 20/12/2012 (M3^M2: 18=23°; 28=20,6°); lower T3: 01/12/2015 (M3^M2 18=7.8°, 28= -1.8°) b. Male patient (08/11/1996) treated with fixed appliances non-extractionally: further uprighting of upper M3 can be expected, together with an improvement in orientation in upper and lower jaw; a close relationship with the inferior alveolar nerve is seen at T3 Left T1: 14/11/2008 (M3^M2: 18=8.8°; 28=11.9°; 38= 55.6; 48=69.1); right T2: 12/07/2013 (M3^M2: 18=25.5°; 28=3.4°; 38=42.2°; 48=43.9°); lower T3: 06/09/2016 (M3^M2: 18=-12.7°; 28=4.1°; 38=8°; 48=13.4°

In Figure 1 cases summarizing the expected evolution of M3 position can be found. Nevertheless, it remains important that the clinician approaches each case individually and considers all discussed parameters.

Finally, it is important to acknowledge certain limitations of the present study. The sample consists of growing individuals only. At T3, most of the M3 studied were still developing. Consequently, their final position remains unknown. However, this is not always clinically relevant, as timely M3 removal diminishes the risk of persistent postoperative morbidity and discomfort.^29^ Also, in (non)-extraction cases, factors such as the use of molar anchorage, initial crowding and the final position of the incisors may have played an additional role. The inherent limitations of two-dimensional radiographs are well-described, such as magnification, loss of information, overlapping, and distortion.^30^ Head positioning errors can aggravate these irregularities. Especially vertical head rotations (5° up and 5° down) influence the angulation of maxillary teeth.^31^ Interpretation of the angular measurements must therefore be done with the greatest care. These problems can be solved by using three‐dimensional radiographs, but the routine use of cone‐beam computed tomography (CBCT) for screening of impacted M3 is not justified taken into account the ALARA principle.

## Conclusions

- Significant further uprighting (M3^M2) of upper M3 can be expected in patients treated with and without HG three years after completion of orthodontic treatment. Significantly more M3 in the HG group presented ideal vertical inclination at T3.
- The use of a HG does not slow down the descent of upper M3 three years after debonding.
- P1-2 extractions do not affect the angulation (M3^M2) of M3 differently three years after orthodontic treatment, although the greatest uprighting of M3 can be expected in NE cases. However, when there is an unfavorable upper and lower M3 orientation or the lower retromolar space appears too small after the end of orthodontic treatment with premolar extractions, significant further improvement cannot be expected and M3 removal should be considered, unlike in NE cases. PM2 extractions lead to an enlargement of the lower retromolar space and to increased chance of M3 eruption three years after completion of treatment.
- The improved vertical position of lower M3 seen in extraction groups after treatment is not maintained three years after. In contrast, PM1-2 extractions accelerate upper M3 eruption in the long term.
- In NE cases rather than in PM1-2 cases where the M3 is already close to the nerve after the end of treatment, it is advisable to consider M3 removal rather than a wait-and-see attitude to prevent IAN damage.
- The mineralization status of M3 is not influenced by the type of treatment in general.
